# Small cell carcinoma of the uterine cervix in pregnancy: A case report and review of the literature

**DOI:** 10.3892/ol.2014.2668

**Published:** 2014-11-04

**Authors:** QING WANG, YI-HONG LIU, LI XIE, WEN-JING HU, BAO-RUI LIU

**Affiliations:** 1 The Comprehensive Cancer Center of Nanjing Drum Tower Hospital, Clinical College of Nanjing Medical University, Nanjing, Jiangsu 210008, P.R. China; 2 Comprehensive Cancer Center of Drum Tower Hospital, Medical School of Nanjing University and Clinical Cancer Institute of Nanjing University, Nanjing, Jiangsu 210008, P.R. China

**Keywords:** cervix, neoadjustchemotherapy, pregnancy, radiotherapy, small cell carcinoma

## Abstract

The occurrence of cervical cancer during pregnancy is extremely rare, particularly small cell carcinoma. Small cell cervical carcinoma (SCCC) is a neuroendocrine tumor with a poor prognosis. This study presents the case of an 18-year-old female with stage IB2 SCCC complicated by pregnancy, who was treated with chemotherapy and radiotherapy. The patient was diagnosed shortly after giving birth, and is the youngest female case to be reported in the world. The patient was treated with cisplatin and etoposide chemotherapy and radiotherapy. Complete remission was achieved following neoadjuvant chemotherapy and radiotherapy, and the patient remains in clinical remission eight months following treatment. Cytological screening, colposcopy and if necessary, biopsy, and selective conization at 14–20 weeks should be considered in the patient evaluation.

## Introduction

Small cell cervical carcinoma (SCCC) is a neuroendocrine tumor with great aggravation. It accounts for only 0.5–3% (1) of cases of cervical cancer and progresses rapidly with early lymphogenous and hemotagenous metastases. The incidence of cervical cancer during pregnancy is ~1/2,000 individuals. Patients with SCCC are more likely to exhibit lymph node metastases and lymph vascular space invasion. Although chemoradiation has been shown to improve survival in non-small cell carcinoma of cervix, the optimal initial therapeutic approach has not been identified in SCCC (2). Standard chemotherapy regimens, such as cisplatin and etoposide, are administered according to the management of small cell lung cancer. The five-year survival rate for SCCC ranges between 0 and 30% (3). The present report describes a case of SCCC in an 18-year-old female, which occurred during pregnancy, and was treated with cisplatin and etoposide chemotherapy and then radiotherapy. Written informed consent was obtained from the patient.

## Case report

**Figure 1:**
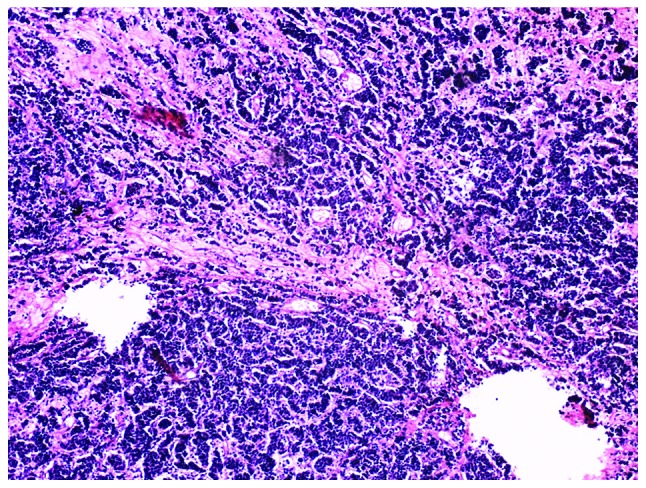
Hematoxylin and eosin-stained section revealing small cell cervical carcinoma with hyperchromatic nuclei and scant cytoplasm (magnification, ×100).

**Figure 2:**
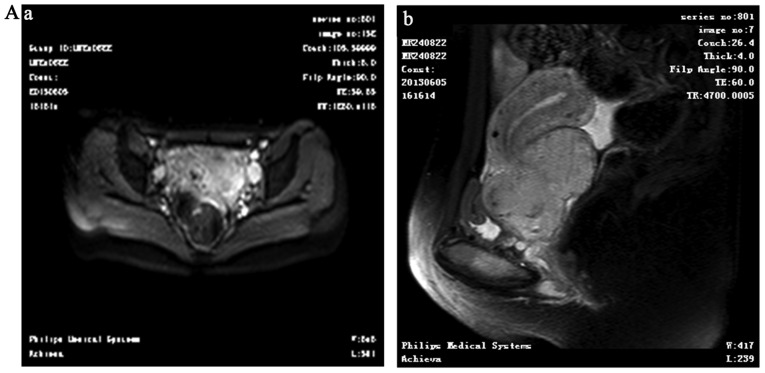

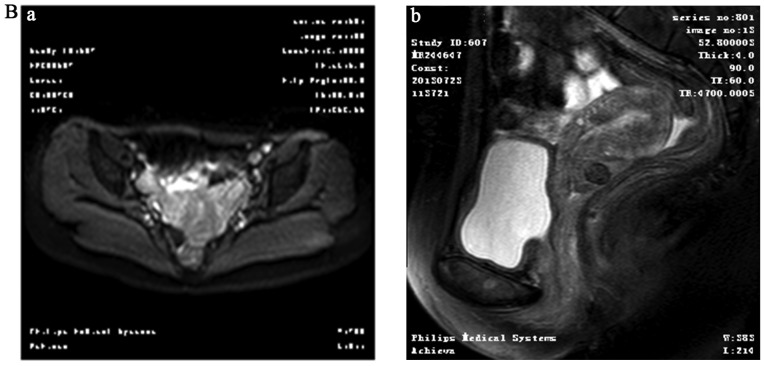

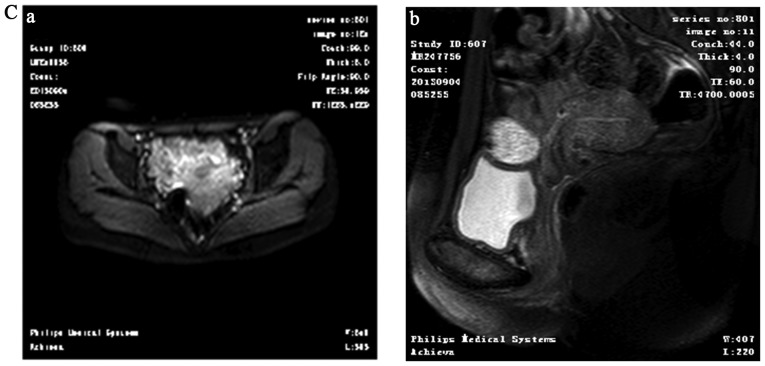
MRI of the pelvis. (A) Prior to treatment, MRI identified an ~6.0×6.0-cm cervical carcinoma, and invasion of the posterior wall of vagina and pelvic lymph nodes. (B) Following two cycles of NACT for six weeks, a decrease in the volume of the vaginal mass was observed. (C) Following four cycles of NACT for 12 weeks, complete clinical remission was achieved. (a) transverse section and (b) median sagittal section. MRI, magnetic resonance imaging; NACT, neoadjuvant chemotherapy.

An 18-year-old Chinese female presented to the Comphrensive Cancer Center of Drum Tower Hospital (Nanjing, China) with intermittent vaginal bleeding of ~50ml/day, for 47 days following delivery. On June 3, 2013, a 6.0×6.0-cm exophytic friable mass was identified in the uterine cervix by colposcopic examination. On histological examination, the mass was diagnosed as SCCC (Fig 1). Immunohistochemistry revealed positive staining for chromogranin A (CgA), cytokeratin, cluster of differentiation (CD)56, synapsin (Syn), Ki67 (50%+) and human papillomavirus (HPV). Magnetic resonance imaging (MRI) confirmed a 5.8×5.7-cm cervical carcinoma and swelling of the lymph nodes in the pelvis (Fig. 2). Based on the histological and radiographical observations (Fig. 1), the tumor was diagnosed as SCCC, and classified as International Federation of Gynecology and Obstetrics (FIGO) stage IB2 (4). Neoadjuvant chemotherapy (NACT) was administered, which consisted of four cycles of treatment with intravenous (i.v) cisplatin (70 mg/m^2^, days 1–3) and i.v etoposide (70 mg/m^2^, days 1–5) for ~9 weeks. Atypical vaginal spotting disappeared following the first cycle of therapy. After the fourth cycle of NACT, MRI revealed a 90% decrease in tumor size when compared with previous MRI scans of the cervical mass and an 80% decrease in size of the lymph nodes (Fig. 2). Three weeks after completing NACT, the patient received 3D intensity-modulated radiation therapy with a total dose of 44 Gy administered to the pelvis and 54 Gy to the pelvic lymph nodes. Image-guided high-dose-rate intracavity brachytherapy at a dose of 25 Gy in combination with low dose Nedaplat (40 mg/m^2^) was administered for eight weeks. MRI showed complete remission had been achieved following radiotherapy. On February 20, 2014, the patient was disease-free without signs of recurrence.

## Discussion

The typical clinical manifestations of SCCC include irregular vaginal bleeding, exudation discharge and severe pain at advanced stages. The diagnosis of SCCC was aided by immunohistochemical staining for common neuroendocrine markers, including Syn, CgA, neuron-specific enolase (NSE) and CD56. Typically, SCCC samples exhibit 100, 96.0, 68.0, 76.0, 40.0, 84.0, 68.0 and 8.0% positivity for NSE, Syn, CD56, CgA, thyroid transcription factor-1, epithelial membrane antigen, cytokeratin and S100 immunohistochemical biomarkers, respectively (5).

At the time of diagnosis, 60.0–82.0% of SCCCs exhibit lymph-vascular space infiltration or pelvic lymph node metastasis (1). SCCC may also rapidly metastasize to the lungs, liver, brain, bones and pancreas. In a previous study, the rate of lymph node metastases in FIGO stage IB1 SCCC was 20%, and in stages more advanced than IB1, the rate was >50% (6). In patients who had relapsed following complete treatment, 67% exhibited hematogenous metastases and 34% exhibited lymphogenous metastases, which indicated a higher probability of hematogenous metastases following first-line treatment. The overall five-year survival rates of 58 patients with SCCC were determined to be ~55–85% for stages IA2–IB1, 25–30% for stage IB2–II and 0–16% for stage III–IV (6). Liao *et al* (1) and Cohen *et al* (7) have reported that the prognosis of SCCC is associated with FIGO stage, tumor size, CgA levels, lymph node metastasis, depth of infiltration and treatment. Patients with FIGO stage I–IIA tumors, tumor size ≤4 cm, negative CgA status, no lymph node metastasis and infiltrating depth of <1/3, exhibited a better prognosis. No significant differences in patient prognosis were observed with regard to NSE, Syn and lymphovascular space invasion. Cohen *et al* (7) found that in a multi-center study of 188 patients, the five-year survival rate for stage I–IIA patients who received a radical hysterectomy was 38.2%, when compared with those who did not undergo surgery was 23.8% (P<0.05). The correlation between HPV infection and the prognosis of SCCC is extremely controversial. One study reported that ~76.2% (64/84) patients were HPV positive (8). HPV infection was associated with better disease-free survival, overall survival (OS) and local control. Vosmik *et al* (9) also demonstrated that patients with HPV positivity and high epidermal growth factor receptor expression had a high probability of achieving complete response (P=0.038 and P=0.044, respectively).

Sood *et al* (10) compared 27 postpartum women, who were diagnosed within six months of giving birth, with 56 women diagnosed during pregnancy. A total of 48–49% of cervical cancers associated with pregnancy were found to be diagnosed within six months of delivery. Of the cervical cancers diagnosed during pregnancy, 89.3% cancers were stage I SCCC, 8.9% were stage II, 1.7% were stage ≥III, whereas the rates were 70.4%, 26.0%, and 3.7% in the women diagnosed after giving birth. Therefore, a higher proportion of early-stage tumors were diagnosed in the pregnant patients (11). Similar to the population of women who are not pregnant, the majority of cases of invasive cervical cancer were squamous cell carcinoma (>80%). Of the remaining cases, the majority were adenocarcinomas, and SCCC was relatively rare.

To date, five cases of pregnant females with SCCC treated with NACT, followed by radical hysterectomy, have been reported. Gestation accelerates the progression of cervical carcinoma due to immunological suppression, high levels of estrogen and an abundant cervical blood supply during pregnancy. In a study by Abeler *et al* (12), 40% patients were HPV-16-positive while 28% were HPV-18-positive (12). All cases of SCCC in pregnant females are shown in Table I. Pregnancy may augment the development of infections, in particular HPV infections. The five-year survival rate of cervical cancer during pregnancy is worse than cervical cancer alone, with rates of 70–78 and 87–92%, respectively.

Small cell carcinoma is hypothesized to be sensitive to chemotherapy and radiotherapy. In the present study, first line systemic chemotherapy to eradicate the potential micrometastases was recommended due to the high probability of metastasis at early stages and the poor prognosis compared with other pathological types of cancer. Platinum-based chemotherapy in combination with pelvic and para-aortic nodal irradiation was administered. A multicenter, collaborative study reported that postoperative chemotherapy improved OS and progression-free survival (PFS) when compared with non-chemotherapy (6). The four-year PFS rate was 65% in the group that received postoperative chemotherapy and 14% without postoperative chemotherapy, and the four-year OS rates were 65 and 29%, respectively. Early stage patients who received post-chemotherapy with the etoposide regimen (cisplatin + etoposide), with or without radiation, exhibited an 83% three-year relapse-free survival, compared with 0% for those who did not receive adjuvant chemotherapy (13), indicating the importance of chemotherapy. No standard treatment regimens have been identified due to the low incidence of SCCC during pregnancy (2). As a result, standard regimens are selected according to the management of small cell lung cancer (3). The method for the treatment of SCCC includes radical surgery (RS) and/or radiotherapy, NACT combined with RS and/or radiotherapy or CCRT (2). Surgery is the first choice at early stages while system chemotherapy is critical at late stages. Katsumata *et al* (14) reported that NACT with BOMP (bleomycin, vincristine, mitomycin, and cisplatin) prior to RS did not improve the OS of patients with stage IB2, IIA and IIB cervical cancer. However, NACT reduced the proportion of patients who received postoperative RT. In a previous study, 10 patients were treated with a regimen of cisplatin, doxorubicin and etoposide followed by radical hysterectomy, pelvic lymphadenectomy and/or radical radiotherapy. The median duration of survival was 28 months (26). In another study, 14 patients with stage IA or IB SCCC received radical hysterectomy alone or in combination with pelvic radiotherapy. A total of 12/14 patients succumbed to the disease within 31 months and the other two patients exhibited recurrence at 38 and 44 months (27). Although the sample sizes were small in these studies, the results indicated that chemotherapy followed by surgery may improve the outcome of patients. However, chemotherapy followed by radiotherapy is controversial (28–30), as a number of phase III clinical trials have demonstrated that chemotherapy followed by radiotherapy does not exhibit any significant survival benefit, however, a different toxicity profile appeared: alopecia (87.9% vs. 4.1%; P<0.001) and neurotoxicity (65.9% vs. 15.6%; P<0.001) were significantly higher in the patients with paclitaxel plus carboplatin followed by radiation. In the present study, the patient was diagnosed with stage IB2 SCCC, with an extensive mass in the cervix and lymph node metastasis in the pelvis. RS was not suitable and the patient underwent four cycles of NACT with cisplatin and etoposide. MRI of the pelvis showed complete remission of the mass in the cervix and lymph nodes. The patient subsequently received radical radiotherapy, due to being unsuitable for RS. A total of four additional cycles of cisplatin and etoposide were administered from February 2014, when the patient exhibited no evidence of relapse.

SCCC in pregnancy must be diagnosed as early as possible. Cytological screening, colposcopy and if required, biopsy, and selective conization at 14–20 weeks gestation should be considered in the patient evaluation (31). The stage of disease, gestational age at diagnosis and patient’s preference must be considered for individualized treatment.

In conclusion, the present study reported a case of SCCC, which occurred during pregnancy. Treatment with NACT and radiotherapy resulted in complete clinical remission. Future clinical trials with a large cohorts are required to determine whether aggressive initial combined therapy, in particular NACT with radiotherapy, may improve the prognosis of advanced SCCC patients.

## Figures and Tables

**Table I:** Cell carcinoma of the cervix during pregnancy: literature review (19–29).

Author (Ref)	Age (years)	FIGO stage	GA at diagnosis (weeks)	NACT. intervals	Treatment	Follow up (months)	Outcome mother	Child
Chun KC *et al* (15)	27	IB1	25	3^*^CDDP 175 mg/m^2^ paclitaxel 75 mg/m^2^	C/S+RH+PLND+PALND	46	DOD	Normal
Smyth EC *et al* (16)	26	IIA	23	3^*^ADM 60 mg/m^2^ CTX 600 mg/m^2^	C/S+4^*^etoposide+pelvic radiation		NED	Normal
Ohwada M *et al* (17)	27	IB1	27	None	C/S+RH+PLND+4^*^etoposide	13	NED	Normal
Leung TW *et al* (18)	26	IB2	31	None	C/S+CCRT (cisplatin 100 mg/m^2^+EP)	14	NED	Normal
Balderston KD *et al* (19)	22	IIA	30	3^*^CDDP 80 mg/m^2^ etoposide 400 mg/m^2^ 2^*^VCR 1.2 mg/m^2^ dactinomycin 300 mg/m^2^ CTX 150 mg/m^2^	Pelvic radiotherapy+4^*^EP	66	NED	Normal
Perrin L *et al* (20)	23	IIA	25	None	C/S; RH,+PLND+adj. chemotherapy (DDP, paclitaxel, etoposide)		DOD	Normal
Chang *et al* (21)	27	IB	36	None	C/S; RH+ PLND+radiotherapy		DOD	Normal
Lojek *et al* (22)	28	IIA	25	None	C/S; PLND; radiotherapy, adj. chemotherapy			
Turner WA *et al* (23)	26	IB	26	None	C/S; RH+PLND, adj. chemotherapy	9	DOD	Normal
Jacobs *et al* (24)	25	IB	10	DDP 50 mg/Kg	RH+PLND, radiotherapy	24	DOD	Normal
Kodousek R *et al* (25)	29	IB	28	None	C/S; RH+PLND, adj. chemotherapy	6	DOD	Normal

FIGO, International Federation of Gynecology and Obstetrics; GA, gestational age; NACT, neoadjuvant chemotherapy; CDDP, cisplatin; ADM, doxorubicin; CTX, cyclophosphamide; VCR, vincristine; DDP, cisplatin; C/S, cesarean section; RH, radical hysterectomy; PLND, pelvic lymph node dissection; PALND, para-aortic node dissection; CCRT, chemoradiotherapy; EP, etoposide; adj., adjuvant; DOD, dead of disease; NED, no evidence of disease.
